# ChatGPT and higher education in Latin America: measuring perceived academic skills

**DOI:** 10.3389/frma.2026.1816902

**Published:** 2026-06-16

**Authors:** Carlos Enrique George-Reyes, Christian A. Romero, Ennio Jesús Merida-Cordova, Enrique Yeguas-Bolivar

**Affiliations:** 1 Universidad Bolivariana del Ecuador, Durán, Ecuador; 2 Universidad de Cordoba, Córdoba, Spain

**Keywords:** academic skills, ChatGPT, generative artificial intelligence, higher education, reliability analysis

## Abstract

The use of generative artificial intelligence tools such as ChatGPT has transformed various academic practices in higher education, generating interest in understanding its perceived impact on the development of students' communicative and analytical skills. This study aimed to validate the Student Perception of ChatGPT instrument. A non-experimental quantitative design with an instrumental and comparative approach was applied to a sample of 2,579 students with prior experience using ChatGPT, from Ecuador, Mexico, Chile, Brazil, Colombia, and Guatemala. The questionnaire included 18 items organized into two key dimensions, communicative skills and analytical skills, measured on a five-point Likert scale. Internal consistency analyses (α = 0.943; ω = 0.944), as well as exploratory and confirmatory factor analyses, were conducted. The results confirmed high reliability and a coherent two-factor structure, with consistent groupings among items related to academic writing, digital communication, critical thinking, and problem-solving. These findings provide empirical evidence of the instrument's validity to measure students' perceptions of ChatGPT's contribution to the development of such skills. It is recommended to conduct longitudinal, mixed-method, and performance-based studies to more precisely examine its actual effect on university learning.

## Introduction

ChatGPT (Chat Generative Pre-Trained Transformer), developed by OpenAI in 2022, is a generative language model recognized for its ability to process information, generate coherent text, and support a variety of academic tasks (Atchley et al., 2024). It is a tool that has been adopted in higher education due to its potential to promote dynamic learning environments, as well as personalized educational experiences (Gill et al., 2024). However, although ChatGPT can improve student learning by supporting activities such as writing, structuring ideas, and retrieving information (Chauke et al., 2024), concerns have been raised regarding academic integrity, the development of critical thinking, and dependence on AI generated content (Essien et al., 2024).

The use of this tool in higher education has had an impact on various dimensions of education such as the development of analytical and communication skills (Peres et al., 2023), as well as enabling students with ideas to facilitate problem solving (Abbas et al., 2024). Its rapid adoption by students and faculty reflects its versatility, as it is increasingly used in routine tasks such as text and code generation, question formulation, article synthesis, research support, as well as in the preparation of assignments, essays, and other academic projects (Kong et al., 2024).

In this regard, the literature review indicates that the pedagogical implications of ChatGPT in higher education contexts have various benefits such as improved access to learning resources and the strengthening of communication skills (Ali et al., 2024). Likewise, it has been stated that the use of ChatGPT fosters the development of analytical skills and facilitates a deeper understanding of content (Abu Khurma et al., 2024). In addition, it has been suggested that this tool can improve academic performance (Essien et al., 2024) and the development of critical thinking skills (Jalil et al., 2023; Wang and Fan, 2025).

However, concerns persist regarding its impact on academic integrity (Escalante et al., 2023), as well as excessive dependence to automate complex academic tasks (Johnson et al., 2024). This may negatively affect the learning process, student performance, and lead to lower memory retention, reduced active engagement, and limited development of cognitive skills (Arugaslan, 2024; Jones, 2023). Likewise, it may encourage plagiarism and compromise transparency and accountability, generating harmful effects on academic integrity (Hosseini et al., 2023).

Nevertheless, when integrated reflectively into curriculum design, these tools can foster constructive feedback and support iterative learning processes (Crompton and Burke, 2023). Therefore, this study seeks to answer the following research question: To what extent does the instrument “Student Perception of ChatGPT” present evidence of validity and reliability to measure the perceived development of communicative and analytical skills among university students in Latin America?

## Generative AI and skill development

The adoption of Large Language Models (LLMs), such as ChatGPT, in higher education has redefined traditional instructional approaches by placing greater emphasis on the development of skills among teachers and students throughout the different stages of teaching learning processes (Escalante et al., 2023). In this regard, Tossell et al. (2024) point out that generative AI systems facilitate the creation of educational content, strengthen student interaction, and personalize instruction according to individual needs. However, their effectiveness lies in the ability to develop skills to simulate human like dialogues and generate coherent text so that the tool can accurately respond to queries in various disciplines (Kasneci et al., 2023).

In this regard, Abualrob (2025) argues that the use of ChatGPT not only facilitates initial reflection processes, but also promotes a more complex “re-reflection,” in which students reinterpret their own learning through iterative interactions with AI. This approach is particularly relevant for teacher education, as it enables the development of metacognitive and critical thinking skills within specific disciplinary contexts. Several studies have documented that the adoption of this tool is mediated by students' perceptions, which combine instrumental benefits with ethical and pedagogical concerns (Alghazo et al., 2025; Moskovich and Rozani, 2025).

In this context, Ibeh et al. (2025) identify that students positively value immediate access to information, support in academic writing, and the personalization of learning; however, they also express concerns related to technological dependence, the loss of cognitive skills, and risks to academic integrity. Similarly, Kostas et al. (2025) show that students recognize both the operational advantages of ChatGPT and its limitations in terms of reliability and autonomous thinking.

The evidence suggests that the impact of ChatGPT on learning is closely linked to usage behaviors as well as ethical and attitudinal factors. Pratita et al. (2025) highlight that interaction patterns with the tool directly influence self-regulated learning processes, fostering more autonomous practices when usage is strategic. Likewise, Rizun et al. (2026) point out that university students' intention to use ChatGPT is shaped by variables such as perceived usefulness, trust in the technology, and the ethical considerations associated with its use.

Likewise, LLMs have driven the automation and semi automation of literature reviews, improving both efficiency and methodological rigor in the systematic extraction of data, while reducing reviewer bias and workload intensity (Bolaños et al., 2024). However, the successful integration of these technologies requires solid human instruction and the development of analytical and communicative skills to ensure that their application is aligned with pedagogical objectives in order to guarantee responsible and effective educational outcomes (Bender, 2024).

The above is limited due to the presence of emerging learning obstacles and pedagogical problems (Holmes and Miao, 2023), such as the lack of familiarity of academics with AI tools (Celik et al., 2022), insufficient technological infrastructure, cultural conflicts, and even issues related to security and privacy protection (Fahimirad and Kotamjani, 2018; Pisica et al., 2023). That is, the digital literacy of both teachers and students, as well as the development of institutional policies, may cause the implementation of this technology not to offer results related to skill development (Guo and Lee, 2023; Kalnina et al., 2024).

In this sense, human beings possess the cognitive capacity to generate original ideas from lived experiences (George-Reyes and Oliva-Córdova, 2025), synthesize complex information (George-Reyes et al., 2024), and apply logical reasoning in diverse contexts (Planer, 2019; Durmuş Sarikahya et al., 2025). This intellectual capacity remains, at least for now, beyond the possibilities of current AI systems (Uno et al., 2024), since, for example, although ChatGPT can generate a 3,000 word academic essay in a matter of minutes, the resulting text is usually the product of recycling, paraphrasing, and recombining existing content, which lacks intentionality and critical reflection (Dergaa et al., 2023).

Consequently, it is essential to implement assessments that make it possible to identify students' perceptions regarding the development of their skills, in order to avoid the false assumption that tools such as ChatGPT automatically promote creativity and critical thinking (Daniel et al., 2023). From this perspective, artificial intelligence tools depend on users' ability to use them in a reflective and critical manner (Alkamel and Alwagieh, 2024), given that, by themselves, they are limited to processing and reproducing previously existing information in digital files, databases, and online sources (Alafnan and Mohdzuki, 2023).

## Methods

## Participants

Students enrolled in undergraduate, master's, or doctoral programs at higher education institutions participated in the study. All participants had prior experience using ChatGPT, which was an essential requirement to complete the questionnaire. This measure ensured that the collected responses reflected perceptions grounded in real experiences with the tool (COVID-19 Social Science Lab, 2024a). Participation was voluntary and anonymous, in compliance with the ethical guidelines of the Declaration of Helsinki (World Medical Association, 2024) and with approval from the corresponding ethics committees (COVID-19 Social Science Lab, 2024b). For this Latin American study, data were collected from six countries: Ecuador (*n* = 1,235), Mexico (*n* = 566), Chile (*n* = 439), Brazil (*n* = 176), Colombia (*n* = 107), and Guatemala (*n* = 56). The first three countries accounted for more than 85% of the regional sample. The diversity of the sample can be observed in Table 1.

**Table 1:** Sociodemographic characteristics of participants from the selected countries.

Participant characteristic	Brazil	Chile	Colombia	Ecuador	Guatemala	Mexico
	176	439	107	1,235	56	566
Student status						
Full-time	99	402	100	1,133	12	399
Part-time	77	37	7	102	44	167
Level of education						
Bachelor's degree	133	411	90	1,228	54	502
Master's Degree	27	25	12	4	0	39
Doctorate	16	3	5	3	2	25
Field of study						
Applied sciences	60	161	102	492	6	168
Arts and humanities	18	62	1	222	2	80
Natural and life sciences	46	32	3	207	45	87
Social sciences	52	184	1	314	3	231
Gender						
Male	81	243	91	649	25	259
Female	95	196	16	586	31	307
Area						
Rural	6	37	0	97	16	18
Urban	15	73	1	167	7	109
Suburban	155	329	106	971	33	439

## Instrument

The instrument used was the *Student Perception of ChatGPT* questionnaire, developed within the framework of the international project *Global ChatGPT Student Survey*, coordinated by the University of Ljubljana (Ravšelj et al., 2024). The dimensions corresponding to sections Q28 and Q29 were selected, which explore perceptions of the impact of ChatGPT on the development of communicative, analytical, and problem solving skills. Both dimensions included a set of nine items each, measured using a five point Likert type scale ranging from 1 = strongly disagree to 5 = strongly agree. The selection of these two dimensions is justified by their relevance to academic training processes in higher education, as they represent essential transversal competencies in university and professional contexts. The selected dimensions and items can be observed in Table 2.

**Table 2:** Questionnaire.

Dimensions	Item	Description
		ChatGPT can improve my...
How much do you agree with the following statements related to ChatGPT's ability to facilitate the development of competence and communication skills?	Q28a	Mastery of academic writing.
	Q28b	Proficiency in professional writing.
	Q28c	Typing proficiency.
	Q28d	Proficiency in the native language.
	Q28e	Proficiency in foreign languages.
	Q28f	Interpersonal communication skills.
	Q28g	Digital communication skills.
	Q28h	Information literacy skills.
	Q28i	Digital content creation skills.
How much do you agree with the following statements related to ChatGPT's ability to facilitate the development of analytical and problem-solving skills?	Q29a	Mastery of arithmetic.
	Q29b	Decision-making skills.
	Q29c	Problem-solving skills.
	Q29d	Analytical skills.
	Q29e	Critical thinking skills.
	Q29f	Creative skills.
	Q29g	Data analysis skills.
	Q29h	Programming skills.
	Q29i	Artificial intelligence literacy skills.

The instrument was subjected to a translation process into Spanish and a linguistic adaptation aimed at ensuring semantic clarity and conceptual equivalence of the items across the six participating countries. This process involved revising the wording to ensure comprehension in different educational and cultural contexts within the region, avoiding ambiguities or technical terms that could be interpreted inconsistently. No substantial modifications were made to the structure or content of the items in order to preserve comparability with the original instrument. In this sense, the present study does not validate the instrument in its entirety, but rather a specific version composed of two selected dimensions, focused on measuring students' perceptions of ChatGPT's contribution to skill development. Therefore, what is being validated is the internal consistency and factorial structure of this partial adaptation within a Latin American context.

## Preliminary analyses

A reliability analysis was conducted, showing high levels of internal consistency (Cronbach's α = 0.943; McDonald's ω = 0.944). The values that the scale would obtain if each item were individually removed were also analyzed. As shown in Table 3, in all cases the coefficients remain above 0.939, indicating that no item compromises the homogeneity of the scale and that its exclusion would not significantly increase the overall reliability of the instrument. This suggests that the items are coherent with the theoretical constructs they represent and contribute in a balanced manner to the measurement of the perceived impact of ChatGPT on the development of communicative (Q28) and analytical (Q29) skills.

**Table 3:** Reliability statistics.

Item	If the item is removed	
	Cronbach alpha	ω McDonald
Q28a	0.941	0.941
Q28b	0.940	0.940
Q28c	0.941	0.941
Q28d	0.940	0.940
Q28e	0.940	0.941
Q28f	0.940	0.941
Q28g	0.940	0.940
Q28h	0.940	0.940
Q28i	0.941	0.941
Q29a	0.941	0.941
Q29b	0.941	0.941
Q29c	0.940	0.940
Q29d	0.939	0.940
Q29e	0.940	0.940
Q29f	0.941	0.941
Q29g	0.939	0.940
Q29h	0.941	0.942
Q29i	0.941	0.941

**Figure 1:**
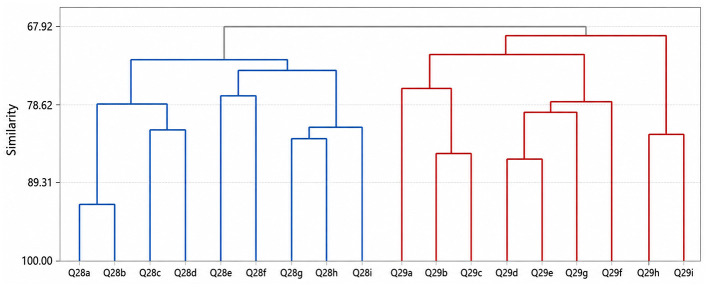
Analysis of hierarchical clusters.

Subsequently, a hierarchical cluster analysis was conducted. Figure 1 shows the progressive grouping of the 18 items. As similarity decreases from 100% to 67.92%, the items merge into clusters with a higher level of heterogeneity. Initially, highly similar pairs are formed, for example Q28a and Q28b with 92.42% similarity, indicating a shared perception among students regarding the impact of ChatGPT on academic and professional writing. Likewise, the groupings largely respect the predefined theoretical dimensions, clearly differentiating between communication skills (blue cluster) and analytical and problem solving skills (red cluster). This analysis adds methodological value by providing complementary evidence of construct validity through the visualization of item similarity patterns, reinforcing the coherence between empirical groupings and the theoretical dimensions of the instrument.

## Results

The first analysis conducted was a principal components analysis. Bartlett's test of sphericity yielded a value of χ^2^ = 30,921, with 153 degrees of freedom and a significance level of *p* < 0.001, indicating that there are significant correlations among the questionnaire items. This justifies the application of data reduction techniques such as factor analysis. As shown in Table 4, after applying a varimax rotation, a differentiation between the communication and analysis dimensions becomes evident. Items related to communicative skills (Q28a–Q28i) show high loadings on the first component, particularly those linked to academic and professional writing (Q28a and Q28b, above 0.76), suggesting that students perceive a more consistent impact of ChatGPT on strengthening these competencies.

**Table 4:** Component loadings.

Item	Component		Uniqueness
	1	2	
Q28a	0.763		0.372
Q28b	0.794		0.320
Q28c	0.713		0.427
Q28d	0.752		0.364
Q28e	0.722		0.400
Q28f	0.613	0.394	0.469
Q28g	0.727		0.383
Q28h	0.672	0.377	0.406
Q28i	0.645	0.347	0.463
Q29a	0.377	0.597	0.502
Q29b		0.733	0.388
Q29c		0.806	0.277
Q29d		0.816	0.263
Q29e		0.786	0.320
Q29f		0.715	0.424
Q29g	0.342	0.733	0.346
Q29h	0.425	0.508	0.561
Q29i	0.473	0.501	0.525

Other items such as Q28c, Q28d, and Q28g maintain significant loadings, confirming the internal coherence of the communicative dimension. In contrast, items associated with analytical and problem solving skills (Q29b–Q29e) cluster with high loadings on the second component, with values ranging from 0.73 to 0.91. This pattern reflects the robustness of a differentiated construct encompassing critical thinking, analysis, and decision making. However, some items such as Q28f present moderate and shared loadings, suggesting intersections between both dimensions and a possible transversal influence of ChatGPT on hybrid competencies that combine communicative aspects with analytical reasoning.

The exploratory factor analysis presented in Table 5 confirms the statistical adequacy of the model by showing a significant chi square test (χ^2^ = 4,365.694; df = 118; *p* < 0.001). The rotation performed allows the identification of two differentiated factors: the first concentrates the highest loadings on items related to analytical and problem solving skills (Q29b–Q29e, with loadings above 0.73), while the second consistently groups items related to written and digital communication (Q28a–Q28g), with loadings ranging from 0.54 to 0.87.

**Table 5:** Factor loadings.

Item	Factor 1	Factor 2	Uniqueness
Q28b	0.872		0.366
Q28a	0.817		0.431
Q28d	0.783		0.414
Q28g	0.738		0.428
Q28e	0.733		0.450
Q28c	0.720		0.486
Q28h	0.635		0.443
Q28i	0.604		0.506
Q28f	0.543		0.507
Q29d		0.906	0.291
Q29c		0.880	0.313
Q29e		0.840	0.372
Q29b		0.737	0.448
Q29g		0.719	0.391
Q29f		0.704	0.496
Q29a		0.510	0.548
Q29h			0.600
Q29i			0.562

The magnitude and distribution of these factor loadings demonstrate that the instrument measures in a differentiated manner two key competencies: communicative and analytical. The coherent grouping of items confirms the structural validity of the questionnaire. In addition, the uniqueness reported in several items (with values between 0.29 and 0.50) supports the robustness of the model, showing that a large proportion of the variance of the items is explained by the extracted factors. Therefore, it can be stated that the instrument accurately captures the dimensions it intends to measure.

**Figure 2:**
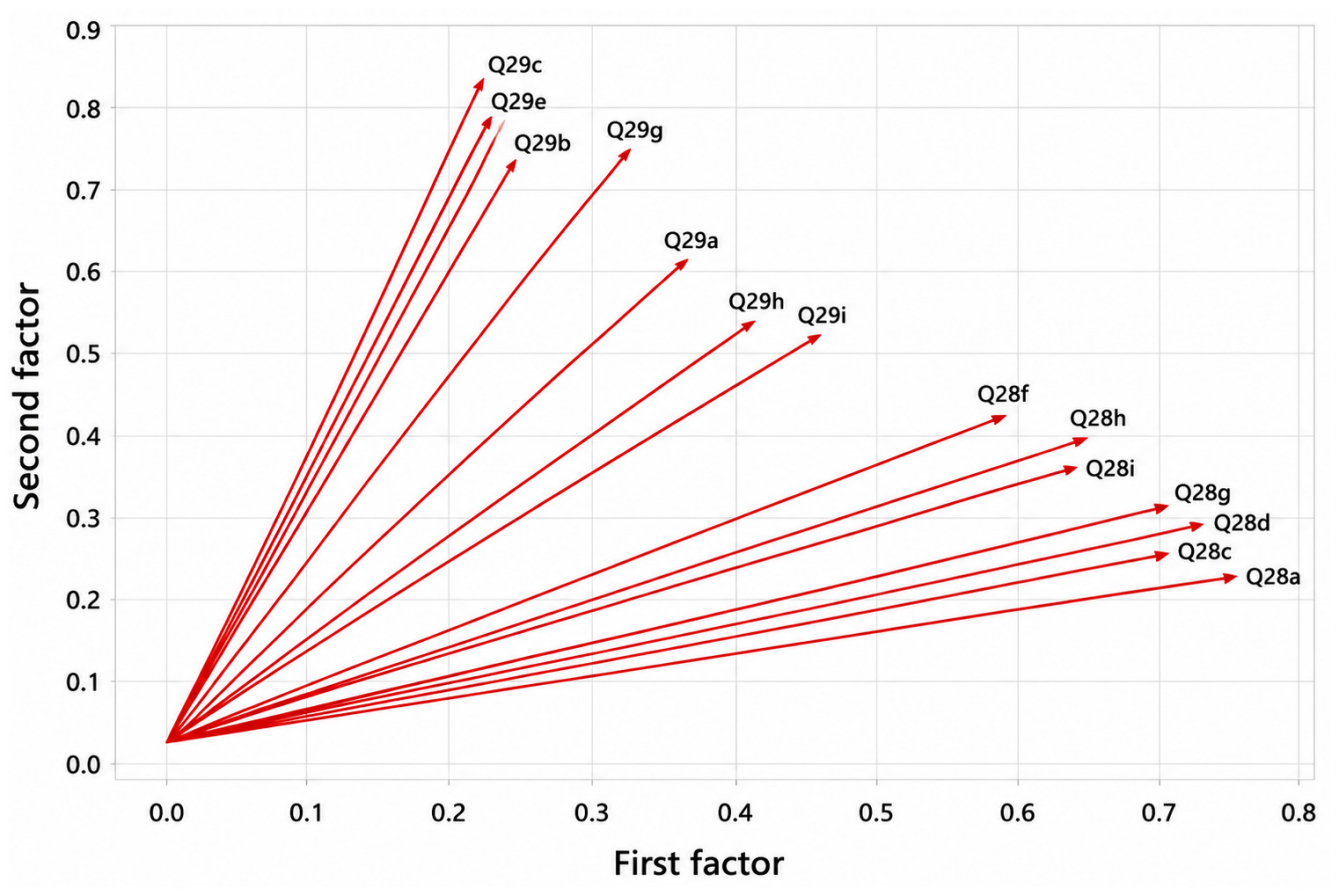
Factor loading plot.

In the factor loading plot shown in Figure 2, items Q28a and Q28b, which assess improvement in academic and professional writing, display high loadings on the first factor (according to the results of the factor analysis in Table 5) and appear close to each other, showing high correlation and low dispersion, which reinforces their internal coherence. Likewise, items Q28g, Q28h, and Q28i, linked to digital communication skills, are concentrated in a common direction, empirically validating their grouping under the second factor. On the other hand, items Q29b, Q29c, Q29d, and Q29e are more strongly oriented toward the axis of the first factor, suggesting a solid factorial structure around critical thinking, problem solving, and quantitative skills. This graphical differentiation is consistent with the previous findings of the principal components analysis and supports the structural validity of the instrument, demonstrating that students' perceptions are not organized randomly, but rather follow logical and coherent patterns aligned with the theoretical constructs of the instrument.

A confirmatory factor analysis was conducted. The baseline model, which assumes total independence among the items, presented a value of χ^2^ = 79,740.444 with 153 degrees of freedom, indicating poor fit. In contrast, the factorial model, which incorporates the expected latent structure of the instrument, substantially reduced the statistic to χ^2^ = 9,725.779 with 134 degrees of freedom and a significance level of *p* < 0.001. This result confirms that the theoretical model provides a much more adequate fit to the observed data, supporting the structural validity of the questionnaire. Table 6 presents robust and statistically significant estimates for the 18 items grouped into two factors. All loading values exceed the recommended minimum threshold of 0.70, with values ranging from 0.829 to 1.191, indicating a strong association between the items and their respective latent factors. The *Z*-values are high and the significance levels (*p* < 0.001) reinforce the robustness of the estimates. In addition, the 95% confidence intervals are narrow and do not cross zero, supporting the precision and stability of the obtained coefficients.

**Table 6:** Confirmatory factor analysis.

Factor	Indicator	Estimate	Typical error	*Z*-value	*p*-value
Factor 1	Q28a	1.000	0.000		
	Q28b	1.025	0.007	147.080	< 0.001
	Q28c	0.829	0.009	93.598	< 0.001
	Q28d	0.876	0.008	111.396	< 0.001
	Q28e	0.853	0.009	95.587	< 0.001
	Q28f	0.847	0.009	90.947	< 0.001
	Q28g	0.899	0.008	109.408	< 0.001
	Q28h	0.896	0.008	108.060	< 0.001
	Q28i	0.857	0.009	94.263	< 0.001
Factor 2	Q29a	1.000	0.000		
	Q29b	1.085	0.014	77.662	< 0.001
	Q29c	1.173	0.014	85.446	< 0.001
	Q29d	1.191	0.014	82.415	< 0.001
	Q29e	1.128	0.014	78.516	< 0.001
	Q29f	1.021	0.014	70.594	< 0.001
	Q29g	1.134	0.014	82.490	< 0.001
	Q29h	1.000	0.015	67.025	< 0.001
	Q29i	1.044	0.015	70.483	< 0.001

**Figure 3:**
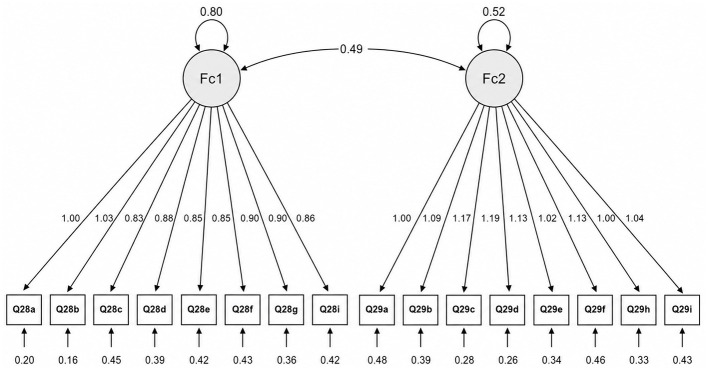
Graph of factor loads.

The structural model represented in Figure 3 confirms a moderate correlation between the factors (*r* = 0.49). The standardized factor loadings of the items range from 0.86 to 1.00 for Fc1 and from 1.00 to 1.19 for Fc2, indicating a strong association between the items and their respective factors. The residual variances, indicated at the bottom of each item, are low, which reinforces the explanatory capacity of the model. Likewise, the variance of the factors (0.80 for Fc1 and 0.52 for Fc2) suggests that a substantial proportion of the variance of the items is explained by the latent constructs.

## Discussion

This study confirms a high level of internal consistency of the instrument, indicating that the items are coherently aligned with the theoretical constructs they are intended to measure. The high values of Cronbach's alpha (α = 0.943) and McDonald's omega (ω = 0.944), which remain stable even when individual items are removed, suggest that the scale reliably captures a homogeneous construct. In line with Crompton and Burke (2023) and Wang and Fan (2025), these results support the importance of psychometrically sound instruments for examining educational phenomena related to artificial intelligence. Importantly, these findings should be interpreted as evidence of the instrument's capacity to consistently measure students' perceptions regarding ChatGPT's contribution to skill development, rather than as direct evidence of actual improvements in communicative or analytical competencies.

Another relevant finding is the clear structural organization of the questionnaire into distinguishable components, which provides empirical support for the proposed theoretical dimensions. The principal components analysis revealed two coherent groupings corresponding to perceived communicative and analytical skills. This differentiation, reflected in the clustering of items related to critical thinking and problem solving on the one hand, and academic and professional writing on the other, suggests that students conceptually distinguish between these domains when evaluating the role of ChatGPT. As noted by Guo and Lee (2023) and Kasneci et al. (2023), a well-defined factorial structure enhances the interpretability of educational instruments; however, in this case, such interpretability is limited to understanding how students perceive the influence of ChatGPT across different skill domains.

Similarly, the exploratory factor analysis confirms the multidimensional nature of the construct being measured, indicating that students' perceptions of ChatGPT's impact are not unidimensional but instead distributed across multiple areas of academic development. The strong loadings of items associated with writing on one factor and those related to analysis and critical thinking on another suggest that participants differentiate between types of perceived benefits. Consistent with Jalil et al. (2023), this multidimensionality allows for a more nuanced representation of the student experience with AI tools. Nevertheless, it is essential to emphasize that these dimensions reflect perceived contributions rather than objectively measured skill acquisition.

The confirmatory factor analysis further supports the adequacy of the proposed model, demonstrating a strong association between observed items and their corresponding latent factors, with statistically significant loadings and an acceptable overall fit. The distinction between communicative and analytical dimensions, along with their moderate correlation (*r* = 0.49), suggests that students perceive these skill areas as related but conceptually independent. Following Crompton and Burke (2023) and Essien et al. (2024), this reinforces the robustness of the measurement model. However, the interpretation of these results must remain within the scope of perception-based data, avoiding causal claims about the actual educational effectiveness of ChatGPT.

The consistency observed in the hierarchical cluster analysis and graphical representations provides additional support for the internal coherence of the instrument. The clear grouping of items into communicative and analytical clusters, as well as the proximity of conceptually related items, reflects stable response patterns among participants. As suggested by Demartini et al. (2024) and Escalante et al. (2023), such visual coherence complements statistical validation by illustrating the structural logic of the instrument. Even so, these patterns should be understood as reflecting how students organize and interpret their experiences with ChatGPT, rather than as direct indicators of measurable gains in academic skills.

## Conclusions

The present study provides empirical evidence regarding the structural validity and high reliability of an instrument designed to assess university students' perceptions of ChatGPT's contribution to the development of communicative and analytical skills in Latin America. The exploratory and confirmatory factor analyses revealed a coherent, differentiated, and theoretically grounded two-factor structure, while the internal consistency coefficients (α = 0.943; ω = 0.944) confirmed the psychometric stability of the questionnaire. These findings support the use of the instrument as a reliable tool for examining how students perceive the role of generative artificial intelligence in relation to key academic skill domains, rather than as a measure of actual skill acquisition or direct educational effects.

This study contributes to the field of education and technology by providing a validated instrument that makes it possible to analyze the pedagogical use of artificial intelligence systems such as ChatGPT from the students' perspective. In addition, by combining advanced psychometric analyses with a comparative cross-country approach, the study broadens understanding of perceived AI-related educational experiences beyond isolated local settings and offers useful evidence for institutional reflection and policy discussions. However, these contributions must be interpreted within the limits of perceptual data, since the findings do not demonstrate that ChatGPT directly improves communicative or analytical skills.

Several limitations should be acknowledged. First, the sample consisted only of students with prior experience using ChatGPT, which excludes the views of non-users or occasional users who may hold different perceptions. Second, the concentration of participants in three countries (Ecuador, Mexico, and Chile) may limit the generalizability of the findings across the broader Latin American region. Third, although robust psychometric analyses were conducted, the cross-sectional design does not allow causal inferences or conclusions about the actual educational effects of ChatGPT on student learning or performance. In addition, if the same sample of 2,579 respondents was used for both the exploratory factor analysis and the confirmatory factor analysis, this should be recognized as a methodological limitation, since the use of a single dataset for both procedures may overestimate model fit and reduce the strength of the confirmatory evidence. Future studies should address this issue by splitting the sample or applying cross-validation procedures.

Future research should apply the instrument in more diverse contexts, including technical institutions, rural settings, and universities with lower levels of digitalization, as well as examine its validity in other linguistic and regional contexts. It would also be valuable to complement perception-based quantitative analyses with qualitative approaches that explore students' experiences and interpretations regarding the use of ChatGPT in specific academic tasks. Future studies should incorporate longitudinal, experimental, or performance-based designs, together with independent validation strategies, in order to determine whether the perceived contributions reported by students are associated with measurable changes in critical thinking, creativity, communication, and academic autonomy.

## Data Availability

The raw data supporting the conclusions of this article will be made available by the authors, without undue reservation.
